# Split-Doa10: A Naturally Split Polytopic Eukaryotic Membrane Protein Generated by Fission of a Nuclear Gene

**DOI:** 10.1371/journal.pone.0045194

**Published:** 2012-10-04

**Authors:** Elisabeth Stuerner, Shigehiro Kuraku, Mark Hochstrasser, Stefan G. Kreft

**Affiliations:** 1 Department of Biology, University of Konstanz, Konstanz, Germany; 2 Department of Molecular Biophysics & Biochemistry, Yale University, New Haven, Connecticut, United States of America; University of Pittsburgh, United States of America

## Abstract

Large polytopic membrane proteins often derive from duplication and fusion of genes for smaller proteins. The reverse process, splitting of a membrane protein by gene fission, is rare and has been studied mainly with artificially split proteins. Fragments of a split membrane protein may associate and reconstitute the function of the larger protein. Most examples of naturally split membrane proteins are from bacteria or eukaryotic organelles, and their exact history is usually poorly understood. Here, we describe a nuclear-encoded split membrane protein, split-Doa10, in the yeast *Kluyveromyces lactis*. In most species, Doa10 is encoded as a single polypeptide with 12–16 transmembrane helices (TMs), but split-*Kl*Doa10 is encoded as two fragments, with the split occurring between TM2 and TM3. The two fragments assemble into an active ubiquitin-protein ligase. The *K. lactis DOA10* locus has two ORFs separated by a 508-bp intervening sequence (IVS). A promoter within the IVS drives expression of the C-terminal *Kl*Doa10 fragment. At least four additional *Kluyveromyces* species contain an IVS in the *DOA10* locus, in contrast to even closely related genera, allowing dating of the fission event to the base of the genus. The upstream *Kluyveromyces* Doa10 fragment with its N-terminal RING-CH and two TMs resembles many metazoan MARCH (Membrane-Associated RING-CH) and related viral RING-CH proteins, suggesting that gene splitting may have contributed to MARCH enzyme diversification. Split-Doa10 is the first unequivocal case of a split membrane protein where fission occurred in a nuclear-encoded gene. Such a split may allow divergent functions for the individual protein segments.

## Introduction

Large polytopic membrane proteins are often generated from smaller membrane proteins by gene duplication and gene fusion events [1]. For example, proteins of the major facilitator superfamily, such as lactose permease, have 12 transmembrane segments (TMs), and these proteins derive from duplication of a six-TM membrane protein gene [2], [3]. The opposite event, i.e. splitting of a polytopic membrane protein by fission of its gene (while maintaining its function), appears to be far more rare. Research on split membrane proteins has focused predominantly on artificially split proteins [4]. Early studies with artificially split sarcoplasmic reticulum adenosine triphosphatase [5] and bacterio-opsin (bacteriorhodopsin) [6], [7] established that separate fragments of split membrane proteins have the potential to assemble into functional proteins (reviewed in [4], [8]).

To date, only a handful of examples of naturally split membrane proteins are known, mostly from bacteria or mitochondria, eukaryotic organelles of α-proteobacterial origin [9]. Mitochondrial genomes are very dynamic, and gene loss and transfer to the nucleus is common [10]. In rare cases, mitochondrial genes have been transferred in pieces, resulting in split proteins that presumably interact *in trans* within mitochondria, fulfilling the same role as the ancestral, intact protein [11]. For example, the *Chlamydomonas reinhardtii* mitochondrial *cox2* coding sequence encoding the essential COX II subunit of cytochrome *c* oxidase had split into two genes, *cox2a* and *cox2b*, both of which subsequently migrated to the nucleus [12]. Fission of the *cox2* gene most likely occurred in the mitochondrial genome [12]. Another example involves the mitochondrial CcmF proteins, which are polytopic membrane proteins with 11 or 13 TMs with similarity to regions of bacterial CcmF proteins [13]. Fission of the *ccmF* gene in plant mitochondria occurred several times independently and at different sites. In each case, the encoded individual protein fragments assemble into a functional multisubunit membrane-protein complex. The molecular mechanisms behind most fission events are only poorly understood. No naturally occurring nuclear gene fissions involving membrane proteins have yet been analyzed experimentally to our knowledge. However, recent bioinformatic analyses imply that such events are not as uncommon as previously supposed and therefore warrant investigation [14].

Here, we report the identification and functional analysis of a naturally split version of the large polytopic membrane protein Doa10 in the milk yeast *Kluyveromyces lactis*. Doa10 has been most thoroughly studied in the yeast *Saccharomyces cerevisiae* where it is expressed as a single polypeptide containing 14 TMs [15], [16]. *Sc*Doa10 is a ubiquitin-protein ligase (E3) of the endoplasmic reticulum (ER)/nuclear envelope [15], [17]. It is involved in both ER protein quality control and degradation of regulatory proteins [18]. Presumptive *DOA10* orthologs are found in almost all sequenced eukaryotic genomes [19], and their products are likely to have topologies similar to that of *Sc*Doa10 [16], [19].

We show that in *K. lactis*, Doa10 is expressed as two separate polypeptides (split-*Kl*Doa10): an N-terminal fragment (Nt-Doa10) consisting of the RING-CH catalytic domain followed by two TMs and a larger C-terminal fragment (Ct-Doa10) encompassing at least 12 TMs. The *DOA10* gene in *K. lactis* is split into two open-reading frames (ORFs) by a 508-bp DNA intervening sequence (IVS). Importantly, the two split-*Kl*Doa10 fragments interact physically and reconstitute a functional ubiquitin ligase. A transcriptional promoter within the IVS mediates expression of the *Kl*Doa10 Ct-fragment. Moreover, four additional species of the *Kluyveromyces* genus for which genomic sequence information is available also contain split-*DOA10* genes, strongly suggesting that a split-Doa10 membrane protein is characteristic of the entire genus. Split-Doa10 is the first example of a split membrane protein whose generation can be traced back unequivocally to the fission of a nuclear gene. The RING-CH family of ubiquitin ligases has diversified in metazoans, with 11 members in humans. Notably, most of these proteins resemble *K. lactis* Nt-Doa10 in having an N-terminal RING-CH domain followed by two TMs. Potentially, the individual fragments of split-*Kl*Doa10 could gain additional individual functions. This incipient evolution might mimic similar events in the expansion and diversification of RING-CH membrane proteins in higher eukaryotes.

## Results

### An intervening sequence (IVS) splits the K. lactis DOA10 coding sequence

**Figure 1:**
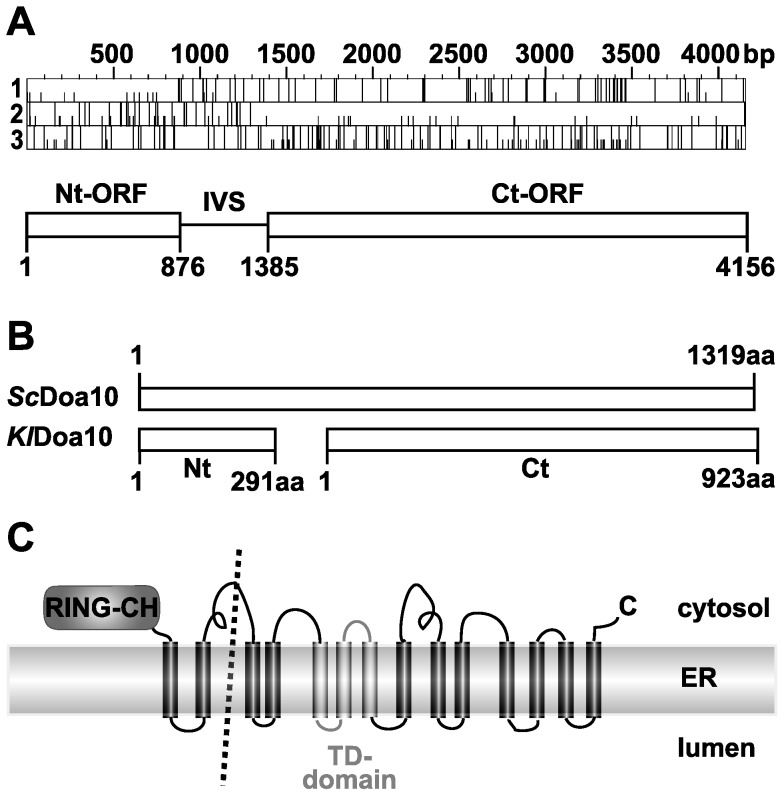
The *Kluyveromyces lactis* Doa10 ORF is split into two ORFs by an intervening sequence (IVS). A. Top, ORF map of the split-*DOA10* locus in *K. lactis* (NCBI: NC_006042.1:2566290-2570445 minus strand) showing the three forward reading frames. Full vertical lines represent stop codons, and half-lines mark AUG (potential start) codons. Bottom, A 508-bp non-coding IVS divides the ORF into an 876-bp N-terminal (Nt-) ORF (NCBI: XM_456313.1) and a 2772-bp C-terminal (Ct-) ORF (NCBI: XM_456312.1). B. Schematic depiction of an alignment of *S. cerevisiae* Doa10 (*Sc*Doa10) protein and the predicted *K. lactis* Nt- and Ct-Doa10 proteins, annotated in NCBI Entrez Gene as hypothetical proteins KLLA0F27709g and KLLA0F27687g, respectively. C. Schematic depiction of the topology of *Sc*Doa10. The predicted split in *K. lactis* Doa10 in the cytosolic loop between TM2 and TM3 is depicted by a dotted line.

The *S. cerevisiae* membrane ubiquitin ligase Doa10 and its orthologs are usually encoded as a single polypeptide [15], [19]. While comparing fungal genomic *DOA10* sequences, we observed that conserved sequences in the putative *K. lactis DOA10* ORF are separated by a stretch with multiple stop codons in all reading frames (Fig. 1A). Generation of a functional Doa10 enzyme from this locus might therefore require pre-mRNA splicing, an internal ribosome entry site (IRES) for expression of the downstream ORF, or transcription of two separate mRNAs. No consensus sites for spliceosomal splicing were detected. The two largest apparent ORFs formed without any splicing are separated by a 508-bp intervening sequence (IVS). The putative N-terminal (Nt-) ORF would encode a 291-residue protein (Fig. 1A, B) with 39% identity to the first 291 residues of *S. cerevisiae* Doa10 (*Sc*Doa10) with the highest similarity in the N-terminal RING-CH domain. The hypothetical *Kl*Doa10 Nt-fragment also contains two transmembrane helices (TMs) corresponding to TMs 1 and 2 of *Sc*Doa10 (Fig. 1C). The C-terminal (Ct-) ORF potentially encodes a 923-residue polypeptide (Fig. 1A, B) with 35% identity to residues 392–1319 of *Sc*Doa10 (Fig. 1C). The highest similarity is found in the TEB4-Doa10-domain (TD-domain) [15], [16], [19].

### Two Doa10 polypeptides are expressed from the K. lactis DOA10 locus

**Figure 2:**
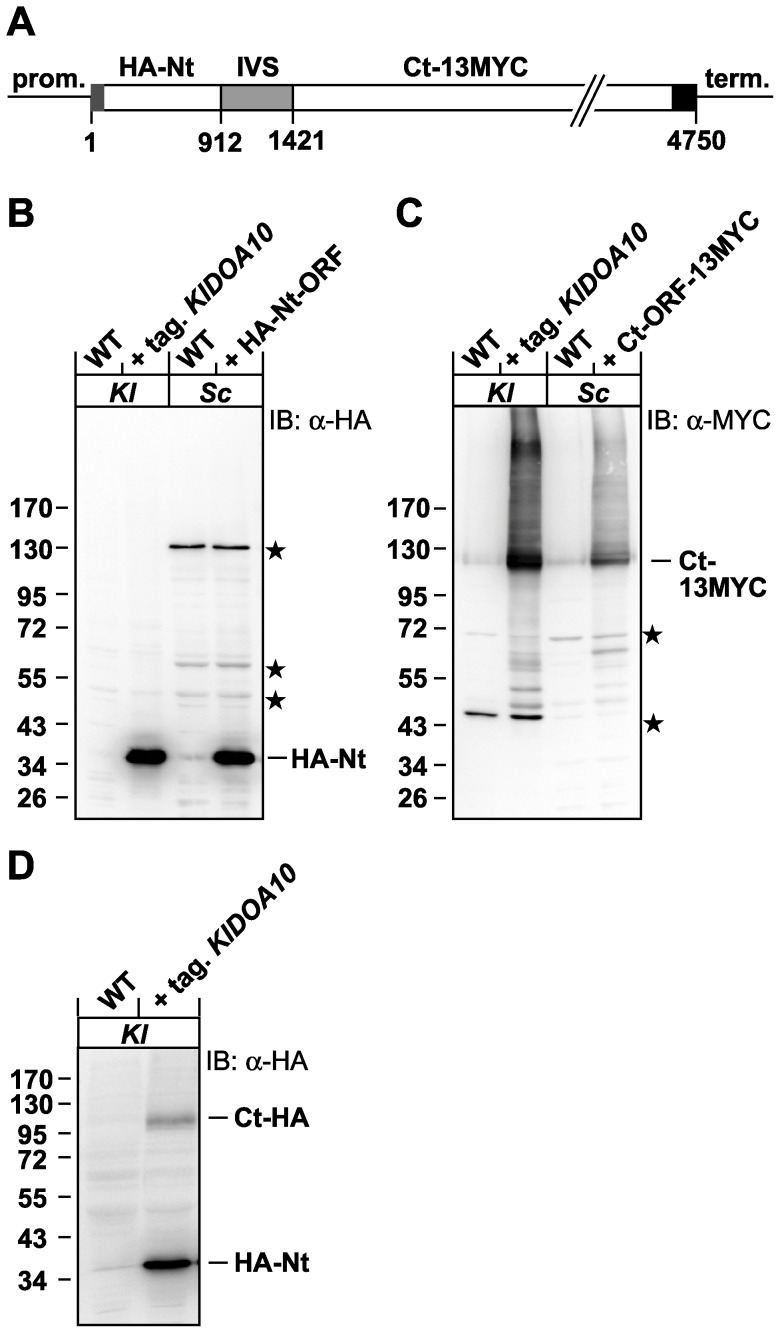
Two Doa10 fragments are expressed from the *DOA10* locus in *K. lactis*. A. A modified version of the *KlDOA10* locus, consisting of the original *DOA10* promoter, an HA-tagged Nt-ORF, the IVS, and a 13MYC-tagged Ct-ORF (and a *CYC1* terminator), was inserted into a promoterless high-copy *K. lactis* plasmid. B. Lysates from WT and tagged-*KlDOA10* plasmid-transformed *K. lactis* cells (*Kl*) were prepared and subjected to anti-HA immunoblotting. A lysate from *S. cerevisiae* cells (*Sc*) expressing the *K. lactis* HA-tagged Nt-ORF (from the strong *S. cerevisiae GPD* promoter in a low-copy plasmid) was loaded for size comparison. Untransformed WT *K. lactis* and *S. cerevisiae* cells served as negative controls. Asterisks, nonspecific bands. C. Anti-MYC immunoblot analysis of WT and tagged-*KlDOA10* plasmid-transformed *K. lactis* cells. A lysate from *K. lactis* 13MYC-tagged Ct-ORF expressing (from the weak *S. cerevisae MET25* promoter in a low-copy plasmid) *S. cerevisiae* cells served as size control, and a lysate from WT *S. cerevisiae* cells as negative control, respectively. Asterisks, nonspecific bands. D. Comparison of HA-tagged *Kl*Doa10 Nt- and Ct-fragment steady state levels by anti-HA immmunoblotting. A modified version of the *KlDOA10* gene locus with HA-epitope tag sequences on both Nt-ORF and Ct-ORF (otherwise as in A) was inserted into a promoterless *K. lactis* low-copy plasmid and transformed into *K. lactis*.

The existence of two partial Doa10 ORFs separated by a 508-bp IVS raised the interesting possibility that *K. lactis* Doa10 is expressed as two separate proteins (“split-*Kl*Doa10”). To directly determine which gene product(s) are expressed from the *K. lactis DOA10* locus, DNA encompassing the entire locus was inserted into a promoterless *K. lactis* high-copy plasmid. An HA-epitope sequence was fused to the 5′ end of the Nt-ORF and a 13MYC-epitope tag was inserted at the 3′ end of the Ct-ORF (Fig. 2A). The resulting plasmid was transformed into *K. lactis* cells, and the expressed gene products were detected by immunoblotting. A ∼36 kDa anti-HA-reactive protein (Fig. 2B, lane 2) and a ∼126 kDa anti-MYC-reactive protein (Fig. 2C, lane 2) were detected. The 36-kDa protein matched the predicted size for the HA-tagged Nt-Doa10 fragment (35.4 kDa). It displayed the same electrophoretic mobility as HA-tagged Nt-Doa10 expressed ectopically in *S. cerevisiae* (Fig. 2B, lane 4). Correspondingly, the ∼126-kDa anti-MYC-reactive fragment exactly matched the predicted size for the 13MYC-tagged Ct-fragment, and its running behavior was identical when expressed ectopically in *S. cerevisiae* (Fig. 2C, lane 4).

To estimate the relative expression levels of the two *Kl*Doa10 fragments, the respective ORFs were tagged with the same HA sequence in a low-copy plasmid carrying the genomic *KlDOA10* gene. Following transformation into *K. lactis* cells, steady-state levels of the HA-tagged fragments in logarithmically growing cells were compared (Fig. 2D). Apparent levels of the HA-Nt fragment were ∼5–10-fold higher than the Ct-HA fragment, although differences in extraction and electrotransfer efficiencies could affect this ratio.

In summary, two proteins are expressed from the *DOA10* locus in *K. lactis*: a 291-residue polypeptide corresponding to an N-terminal fragment of *Sc*Doa10 and a 923-residue polypeptide corresponding to the C-terminal segment of *Sc*Doa10. We therefore refer to the product of the *KlDOA10* locus as “split-*Kl*Doa10”.

### The KlDoa10 fragments interact physically to form a functional ubiquitin ligase

**Figure 3:**
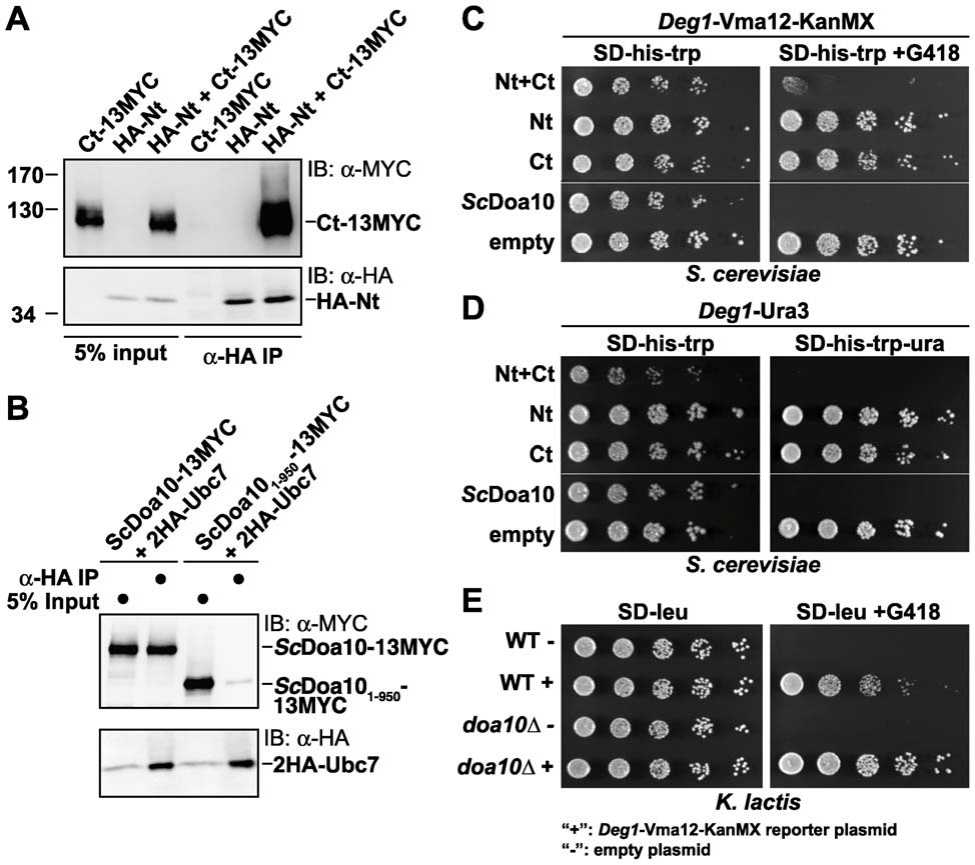
*Kl*Doa10 Nt- and Ct- fragments interact and form a functional ubiquitin ligase. A. Co-immunoprecipitation (co-IP) analysis of digitonin-solubilized microsomes. HA-tagged Nt-*Kl*Doa10 and 13MYC-tagged Ct-*Kl*Doa10 proteins were either individually expressed or co-expressed in *K. lactis doa10Δ* cells, and HA-Nt-*Kl*Doa10 was precipitated with anti-HA agarose beads. Precipitates were analyzed by immunoblotting with anti-MYC or anti-HA. B. Control demonstrating specificity of the co-IP protocol. HA-epitope-tagged Ubc7 was precipitated with an anti-HA antibody from digitonin-solubilized microsomes derived from *S. cerevisiae* cells expressing MYC-tagged full-length Doa10 or a C-terminally truncated derivative, Doa10_1–950_. Precipitates were analyzed by immunoblotting with anti-MYC or anti-HA antibodies. Co-precipitation is only observed for full-length Doa10. The strain (MHY3000) was made by T. Ravid and M.H. (unpublished). C. Co-expression of *Kl*Doa10 Nt- and Ct-fragment reconstitutes Doa10 E3 ligase activity toward the model membrane substrate *Deg1*-Vma12-KanMX in an *S.cerevisiae doa10Δ* strain. Serial dilutions of *S. cerevisiae* transformants expressing the indicated epitope-tagged *Kl*Doa10 fragment(s), FLAG-tagged Nt and 13MYC-tagged Ct, were spotted onto minimal plates lacking histidine and tryptophan (SD-his-trp) with or without G418 added. *Sc*Doa10, *S. cerevisiae* (*doa10Δ*; *Deg1*-Vma12-KanMX) strain expressing functional *Sc*Doa10 from a plasmid served as a control. Upper and lower panels are from the same plate. D. Co-expression of *KlDoa10* Nt- and Ct- fragment reconstitutes E3 ligase activity toward the soluble model substrate *Deg1*-Ura3 in *S.cerevisiae doa10Δ* cells. Serial dilutions of the indicated transformants were spotted onto SD-his-trp and SD-his-trp-ura plates. Upper and lower panels are from the same plate. E. Endogenous split-*Kl*Doa10 is active toward the model membrane substrate *Deg1*-Vma12-KanMX. Serial dilutions of WT or *doa10Δ K. lactis* cells transformed with a *Deg1*-Vma12-KanMX reporter plasmid (+) or an empty control plasmid (−) were spotted onto SD-leu and SD-leu+G418 plates.

We tested whether the two *Kl*Doa10 fragments associate with one another. HA-Nt-*Kl*Doa10 and Ct-*Kl*Doa10-13MYC proteins were expressed from separate plasmids in *K. lactis doa10Δ* cells, and the HA-Nt fragment was immunoprecipitated from digitonin-solubilized microsomal protein extracts with anti-HA agarose beads (Fig. 3A). The Ct-*Kl*Doa10-13MYC protein was efficiently precipitated but only in the presence of the HA-Nt fragment. As a control for the completeness of the digitonin solubilization, a set of control anti-HA immunoprecipitations using the same conditions was done with *S. cerevisiae* expressing tagged *Sc*Doa10 and its cofactor Ubc7 (Fig. 3B). Full-length *Sc*Doa10-13MYC co-precipitated with HA-tagged Ubc7, whereas a C-terminally truncated *Sc*Doa10 variant (*Sc*Doa10_1–950_-13MYC) known not to binding tightly to Ubc7 [19] showed only minimal interaction under our conditions. Taken together, these results show that Nt- and Ct-fragments of split-*Kl*Doa10 physically interact.

We next determined if the two *Kl*Doa10 fragments reconstituted a functional ubiquitin ligase using two model substrates, one a membrane substrate, *Deg1*-Vma12-KanMX (*Deg1*-VK) [19], and the other a soluble protein, *Deg1*-Ura3 [20]. The *Deg1* moiety functions as a degradation signal that is specifically recognized by Doa10 [15].Vma12 is an ER-membrane protein with two TMs and cytosolically exposed termini [21], and the KanMX moiety confers resistance to the antibiotic G418. Doa10 activity toward *Deg1*-VK is readily gauged by growth on G418 plates. A functional Doa10 pathway causes rapid *Deg1*-VK degradation, leaving cells sensitive to G418, whereas impaired *Deg1*-VK degradation renders them resistant (Fig. 3C). *S. cerevisiae doa10Δ* cells harboring a chromosomal *Deg1*-VK reporter were transformed with plasmids that expressed N-terminally FLAG-tagged Nt-*Kl*Doa10 and C-terminally 13MYC-tagged Ct-*Kl*Doa10. Cells co-expressing both *Kl*Doa10 fragments (“Nt+Ct”) grew significantly slower on G418 plates than cells expressing only one of the two fragments or neither (Fig. 3C). The *Kl*Doa10 proteins were properly expressed in each transformant (Fig. S1A).

Activity of split-*Kl*Doa10 toward the soluble substrate *Deg1*-Ura3 was tested by growth on medium lacking uracil (SD-ura). Ura3 is required for uracil biosynthesis, and rapid degradation of *Deg1*-Ura3 impairs cell growth on this medium when *Deg1*-Ura3 is the only source of Ura3 activity. Co-expression of the two epitope-tagged *KlDoa10* fragments in *S. cerevisiae doa10Δ* cells containing a chromosomal copy of the *Deg1*-*URA3* strongly inhibited growth on SD-ura, as is seen for cells expressing *Sc*Doa10 (Fig. 3D). Co-expression of the two *KlDoa10* fragments was required for this effect (For unknown reasons, co-expression of the two *KlDoa10* fragments resulted in a mild growth defect of these cells even when uracil was present). Expression of the corresponding *Kl*Doa10 fragment(s) was verified by Western blotting (Fig. S1B).

Lastly, we investigated whether endogenous split-*Kl*Doa10 is active toward *Deg1*-VK in *K. lactis* (Fig. 3E). Wild-type (WT) *K. lactis* cells containing the *Deg1*-VK expression plasmid grew significantly slower in the presence of G418 than did the *doa10Δ* transformants (Fig. 3E), indicating E3 activity of endogenous split-*Kl*Doa10. Taken together, the results demonstrate that the *Kl*Doa10 Nt- and Ct-fragments interact to form a functional E3 ligase. Neither of the two split-*Kl*Doa10 fragments displays detectable Doa10 E3 activity by itself. While these assays do not measure ubiquitin ligase activity per se, the *S. cerevisiae* Doa10 protein is a well-established E3 ligase, and the substrates tested here are known Doa10 ligase-dependent substrates. Therefore, one can be confident that the complementing *K. lactis* Doa10 fragments are reconstituting a functional E3 ligase since ligase activity is necessary for degradation of these substrates.

### The IVS contains a transcriptional promoter

Expression of two separate proteins from the *DOA10* locus in *K. lactis* raised the question of the mechanism of Ct-fragment expression. As the IVS separating the Nt- and Ct-ORFs bears no obvious similarity to sequences in the GenBank database, no function could be inferred from its sequence. Since the production of two *Kl*Doa10 proteins of the expected size argued against pre-mRNA splicing, we imagined that the IVS could either act as an IRES for translation of the Ct-fragment or as a transcriptional promoter for generation of a second mRNA encoding the Ct protein. In the former case, a single bicistronic *Kl*Doa10 mRNA originating from the *KlDOA10* promoter upstream of the Nt-ORF would be predicted. In contrast, two separate mRNAs would be generated from the *KlDOA10* locus if the IVS functioned as a transcriptional promoter.

**Figure 4:**
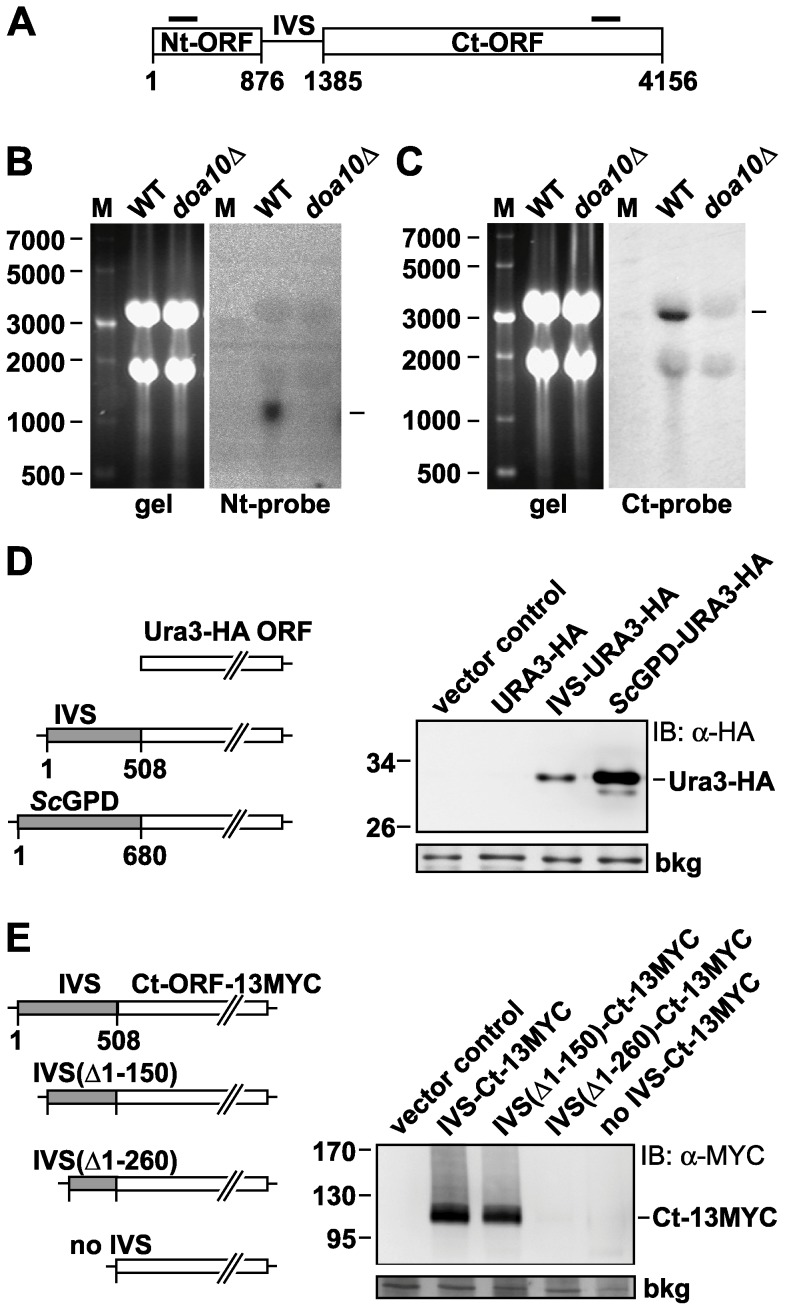
A transcriptional promoter in the *KlDOA10* IVS. A. Schematic representation of the *KlDOA10* gene consisting of the Nt-ORF, the IVS and the Ct-ORF. The annealing sites of the Nt and Ct ORF-specific hybridization probes are depicted as black horizontal bars. B. Northern blot analysis of *KlDOA10* transcripts with a *KlDOA10* Nt-ORF specific probe (Nt-probe). Total RNA was analyzed from WT and *doa10Δ K. lactis* cells. A picture of the ethidium bromid-stained agarose gel before transfer to membrane is shown on the left (gel). The PhosphorImager-scanned image on the right was derived from a 3 d exposure of the Nt probe-hybridized membrane. M, RNA size markers (in nts). The horizontal bar on the right marks the position of the ∼1200-nt *KlDOA10* Nt-ORF-specific transcript. C. Northern blot analysis of *KlDOA10* transcripts with a *KlDOA10* Ct-ORF specific probe (Ct-probe). Northern blotting was done as in Fig. 4B except a [^32^P]-labeled Ct-probe was used for hybridization. The hybridization image is from a 7 d film exposure (Ct-probe). The horizontal bar marks the position of the ∼3200-nt *KlDOA10* Ct-ORF-specific transcript. D. IVS promoter activity in the absence of other *KlDOA10* sequences. *Left*: Schematic representations of the IVS-URA3-HA and the *Sc*GPD-URA3-HA sequences that were inserted into a promoterless *K. lactis* low-copy plasmid. The constructs were transformed into *K. lactis* cells, and expression of the Ura3-HA reporter was examined by anti-HA immunoblotting (*right).* A nonspecific background band (bkg) served as a loading control. E. Mapping of the 5′ border of the promoter contained within the IVS. *Left*: Schematic representation of the IVS-Ct-ORF-13MYC sequence and 5′-truncated versions of the IVS that were cloned into a promoterless *K. lactis* low-copy plasmid. The different constructs were transformed into *K. lactis* cells and expression of the Ct-13MYC fragment was examined by anti-MYC immunoblotting (*right).* A nonspecific background band (bkg) served as a loading control.

To distinguish these models of IVS function, we analyzed endogenous *KlDOA10* transcripts by Northern blotting with Nt- and Ct-ORF specific probes (Fig. 4A). In total RNA from WT *K. lactis* cells, a single ∼1200-nucleotide (nt) transcript was detected with the Nt-probe (Fig. 4B), consistent with an mRNA comprising the Nt-ORF including 5′- and 3′-UTRs (terminating somewhere within the IVS) and a poly(A) tail. This transcript was *KlDOA10*-specific as it was not present in total RNA from *K. lactis doa10Δ* cells. A single ∼3200-nt RNA was detected in the WT sample with the Ct-ORF specific probe (Fig. 4C), consistent with a transcript originating in the IVS and containing the complete Ct-ORF as well as a 3′-UTR and poly(A) tail. The ∼3200-nt transcript was not detected in *doa10Δ* cells. The existence of two distinct *KlDOA10* transcripts of roughly the size expected for separate expression of the Nt-ORF and Ct-ORF strongly suggests that the IVS functions as a transcriptional promoter.

For a direct test of *KlDOA10* IVS promoter activity, we inserted the 508-bp IVS in front of a *URA3-HA* reporter gene in the context of a promoterless *K. lactis* low-copy plasmid. A similar construct containing the *S. cerevisiae GPD* promoter was analyzed in parallel for comparison. Following transformation into *K. lactis* cells, lysates were prepared and Ura3-HA expression was examined (Fig. 4D). The IVS itself was sufficient to drive reporter expression (*IVS*-*URA3-HA*) and expression was strictly dependent on the presence of the IVS. The strong *S. cerevisiae GPD* promoter led to a ∼5-fold higher expression of the reporter compared to the *KlDOA10* IVS. Using a 13MYC-tagged Ct-*Kl*Doa10 reporter for IVS promoter activity, we could show that the first 150 bp of the IVS were dispensable, but deletion of the first 260 bp abrogated promoter activity (Fig. 4E).

We conclude that the *K. lactis DOA10* locus expresses two mRNAs, an upstream transcript encoding the Nt-ORF, and a downstream transcript driven by a promoter element within the IVS encoding the Ct-ORF.

### Fission of the DOA10 gene is a characteristic feature of the Kluyveromyces genus

**Figure 5:**
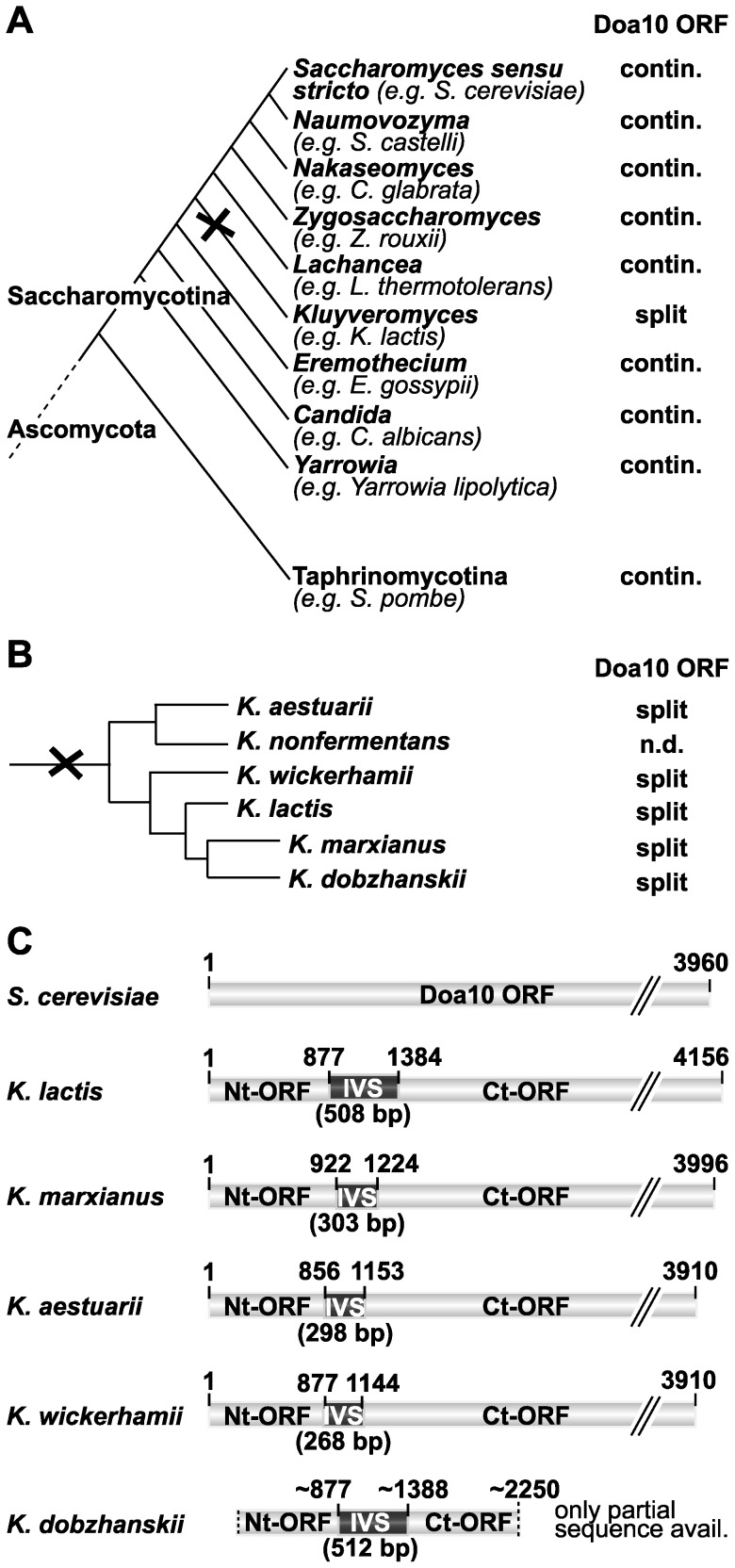
Split-Doa10 is a characteristic feature of the *Kluyveromyces* genus. A. Phylogenetic tree of yeast/Saccharomycotina. The presence or absence of an intervening sequence (IVS) within the *DOA10* locus is indicated for each genus. *DOA10* fission occurred at the root of the *Kluyveromyces* genus (black cross). B. Phylogenetic placement of species currently assigned to the genus *Kluyveromyces* (adapted from [23]). The experimentally verified presence of an intervening sequence (IVS) disrupting the Doa10 ORF is indicated. *DOA10* fission at the root of the genus is symbolized by a black cross. C. Schematic comparison of genomic *DOA10* ORFs of *S. cerevisiae* and *Kluyveromyces* species. Numbers above each IVS indicate the first and last nucleotide of the IVS, respectively. For further information on gene loci listed in (A–C) and corresponding gene products see Table S1 and Table S2.

Sequence analysis has indicated that Doa10 orthologs from fungi and a broad range of other eukaryotes are expressed as single large polypeptides (Fig. 5A and Table S1). This suggests that a single large polytopic protein was encoded by the *DOA10* locus in the last common eukaryotic ancestor (LECA) [19]. Thus, the *K. lactis* split-*DOA10* gene very likely reflects a relatively recent fission of the once continuous *DOA10* ORF in the nuclear genome of a *K. lactis* progenitor.

To help locate this progenitor, we determined whether split-Doa10 is also present in other members of the genus *Kluyveromyces*, which currently counts six members (*K. lactis*, *K. marxianus*, *K. aestuarii*, *K. wickerhamii*, *K. dobzhanskii* and *K. nonfermentans* (Fig. 5B and [22], [23])). When this study was initiated, *K. lactis* was the only *Kluyveromyces* species with a completely sequenced genome [24]. In addition, random sequence tags from an exploratory genome-sequencing project with partial genome coverage is available for *K. marxianus* [25]. We succeeded in amplifying and sequencing the complete *K. marxianus DOA10* locus. It contains a 303-bp IVS at a similar position as in *K. lactis DOA10* (Fig. 5C and Table S2). Furthermore, amplification and sequencing of part of the *K. dobzhanskii DOA10* locus revealed the presence of a 512-bp IVS (Fig. 5C and Table S2). In the course of the current work, complete (but not yet annotated) genome sequences of *K. aestuarii* and *K. wickerhamii* became available [26]. We identified and analyzed the *DOA10* locus in these two species and found that both also contain a split-*DOA10* gene (Fig. 5C and Table S2). Notably, the IVSs of these different *Kluyveromyces DOA10* genes vary considerably in length and sequence (see Discussion).

Given this sequence diversity, we tested one of the shortest IVS elements, the 303-bp IVS from *K. marxianus*, for promoter activity. In Nothern blots of total RNA, two *KmDOA10* specific transcripts were detected, a ∼1400-nt species seen with an Nt-ORF-specific probe and a ∼3100-nt RNA detected with a Ct-ORF probe (Fig. S2B, C), suggesting an internal promoter driving expression of the Ct-ORF mRNA. Consistent with this, the 303-bp *Km*IVS was also sufficient to drive reporter gene expression in *K. lactis* (Fig. S2D).

Collectively, the presence of an IVS in the *DOA10* locus of all five tested *Kluyveromyces* species strongly suggests that a two-subunit Doa10 enzyme is a common feature of the entire genus, and points to a gene splitting event occurring near the root of the genus (Fig. 5A, B).

## Discussion

Functional splitting of a membrane protein into multiple integral membrane fragments by fission of its gene appears to be a fairly rare event in evolution. All previously described cases of naturally split membrane proteins are from bacteria or bacterially derived eukaryotic organelles. Here we have identified split-*DOA10* in the yeast genus *Kluyveromyces* as the first case, to our knowledge, of a split polytopic membrane protein where the split unambiguously occurred in the context of a nuclear genome. Below we discuss how split-Doa10 is expressed, advantages potentially associated with the split, and possible gene fission mechanisms.

Likely orthologs of the *DOA10* gene are present in the majority of sequenced eukaryotic genomes and are virtually always predicted to encode Doa10 as a single large polypeptide [19]. By contrast, for all tested members of the genus *Kluyveromyces*, Doa10 is expressed as two separate fragments, with the split occurring in the cytosolic loop between TM2 and TM3 (Fig. 1 and Fig. 2). The upstream fragment of split-*Kl*Doa10 includes the N-terminal RING-CH domain and two TMs, and the Ct-fragment spans the region from TM3 to the end, including the conserved TD-domain. The two fragments physically associate in *K. lactis* and can reconstitute an active ubiquitin ligase (Fig. 3). Exactly which regions of the two subunits are important for these protein-protein interactions remains to be determined. Compared with the intact *Sc*Doa10 protein, a stretch of ∼100 residues within (the split) loop 2 is apparently missing in split-*Kl*Doa10 (schematically depicted in Fig. 1B). However, loop 2 in all Doa10 proteins is of low complexity and is predicted to include a high fraction of intrinsically disordered sequence. Thus, it is difficult to align sequences between TM2 and TM3 of the various Doa10 orthologs.

The IVS splitting the *KlDOA10* ORF contains sequences that function as promoter elements. Two distinct Doa10 transcripts were detected, one expressing the Nt-ORF and the other the Doa10 Ct-ORF, and the IVS by itself can drive expression of a reporter gene (Fig. 4). Promoter activity could also be demonstrated for the *K. marxianus DOA10* IVS (Fig. S2). The fact that the IVS sequences of the various *Kluyveromyces* species are not readily aligned, whereas Nt- and Ct-ORF coding sequences are, suggests that much of the IVS is not under strong evolutionary constraints. No shared motifs between the different *Kluyveromyces* IVS elements have been identified yet. Further work will be necessary to fully define these newly identified promoters.

An obvious question regarding the splitting of the *DOA10* ORF in the genus *Kluyveromyces* is whether it confers any selective advantage over expression as a single long polypeptide. Potentially, the event represents a “frozen accident” of no selective value that has persisted through subsequent evolution into different *Kluyveromyces* species because of the improbability of reversing the interruption of the originally continuous *DOA10* ORF (see below). Alternatively, expression as two mRNAs potentially allows for subtle changes in the regulation of Doa10 activity. For example, interaction of the two subunits might be controlled by post-translational modification, and this may allow more rapid increases or decreases in Doa10 ubiquitin ligase activity, such as in response to environmental stresses that increase misfolded protein levels in the ER. Another possibility is that the two Doa10 subunits might accrue additional individual functions, either alone or together with other proteins. For instance, the Nt-ORF, which has the catalytic RING-CH domain and two TMs, might gain the ability to target additional membrane-associated substrates distinct from those recognized by the full Doa10 enzyme.

We previously proposed that Doa10 might represent a combination of a ubiquitin ligase and a channel or extraction pore for retrotranslocation of its membrane substrates from the ER and nuclear envelope membranes [15], [16], [19]. Intriguingly, the N-terminal fragment of split-Doa10 closely resembles viral MIR (Modulator of Immune Recognition) proteins and most mammalian MARCH (Membrane-Associated RING-CH) proteins, which are also composed of a cytosolic N-terminal RING-CH domain and two TMs [27]; MARCH6/TEB4, by contrast, is the ortholog of yeast Doa10 and has 14 predicted TMs [16]. These MARCH proteins function in various cellular processes, often in the ubiquitylation of surface receptors at the plasma membrane to trigger their endocytosis (reviewed in [28], [29]). It is possible that the *Kluyveromyces* split-Doa10 Nt-fragment might act as an E3 ligase activity without the Ct-fragment. The apparent excess of the Nt-fragment over the Ct-fragment (Fig. 2) would be consistent with a subpopulation of the Nt-fragments being deployed as a ubiquitin ligase independent of the Ct-fragment. Similarly, the Ct-fragment could in principle function as a pore/channel on its own or together with proteins other than the Doa10 Nt-fragment. Moreover, the natural occurrence of a split in the Doa10 protein provides a unique opportunity for mapping specific functions, such as E2 binding, to individual E3 domains.

The site at which Doa10 in *Kluyveromyces* species was split is most likely not random. As noted, loop 2 where the split occurred is a region of low sequence complexity, which would be predicted to be more permissive toward DNA insertion and/or sequence divergence. Each of the two fragments can insert into the ER membrane without the other (unpublished, E.S. and S.G.K.) and is metabolically stable (Fig. S3). These observations indicate that proper biogenesis and folding of the fragments do not require the respective partner fragment although a more subtle folding deficiency of individual subunits in the absence of the respective partner cannot be completely ruled out. In light of *K. lactis* split-Doa10 it seems conceivable that fission of intact *DOA10*, presumably after duplication, and subsequent loss of the Ct-fragment encoding sequence, has contributed to the diversification of the smaller (2-TM) MARCH proteins.

**Figure 6:**
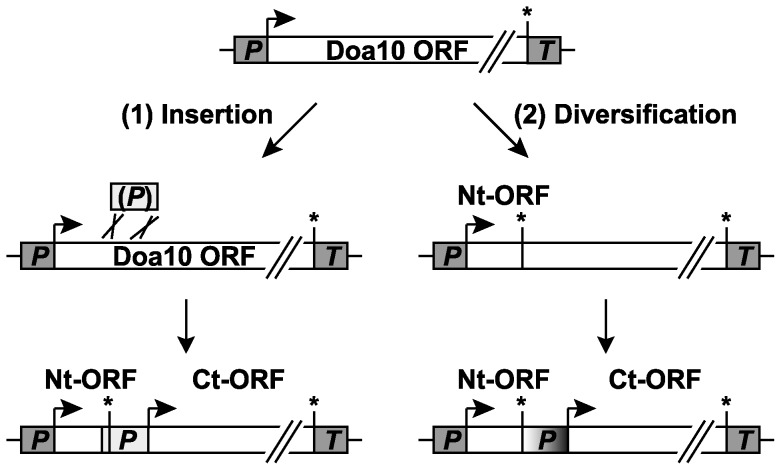
Possible gene fission scenarios for *Kluyveromyces DOA10.* Fission of the Doa10 ORF might have occurred by either of the two following ways: (1) Splitting by insertion of a foreign sequence into the *DOA10* gene by a recombination event. The Doa10 ORF is disrupted by integration of the foreign sequence (light grey box) whereby a stop codon is created leading to the Nt-ORF and the Ct-ORF. The Ct-ORF starts close to the 3′ end of the inserted sequence (as depicted) or, alternatively, within the inserted sequence (not depicted). The foreign sequence might have already had promoter activity (indicated by “(P)”) before insertion or might have acquired it later. (2) Splitting by internal diversification of the Doa10 coding sequence. The acquisition of a stop codon, e.g. by point mutation, resulted in generation of the Doa10 Nt-ORF. The region downstream of the newly created stop codon (shaded in grey) might have displayed (residual) promoter activity or alternatively, might have subsequently evolved into a promoter driving expression of the downstream Ct-ORF. See main text for details. P, promoter; T, terminator; the beginning of an ORF is depicted with an arrow, its end with an asterisk.

Another interesting question regarding split-Doa10 is the mechanism of gene fission. The high sequence divergence of the IVS in different *Kluyveromyces* species complicates this issue. We envision two general scenarios, although additional more complicated ones can be imagined (Fig. 6). In one, gene fission occurred by insertion of a DNA fragment with promoter activity into the *DOA10* ORF. In the other, internal diversification of the *DOA10* coding sequence led eventually to two ORFs expressed from separate mRNAs. The second scenario requires the generation of a functional promoter that drives expression of the Ct-ORF, which might seem less likely. However, it is possible that the loop 2-coding segment of single-ORF *DOA10* orthologs evolved from an earlier sequence where it had functioned as a transcriptional promoter. Transformation of the sequence back to an active promoter might therefore be less difficult than its *de novo* generation. Such a “cryptic promoter” might be more plausible if the long single-ORF *DOA10* orthologs arose from an earlier event that fused a “RING-CH+2TM protein” gene in-frame with a “12-TM protein” gene, with the latter including its promoter. RING-CH+2TM protein genes have recently been uncovered in prokaryotes and might represent an ancestral form [30], [31]. Notably, the combined length of the two *DOA10* ORFs plus the IVS in *Kluyveromyces* species is similar to that of the single longer ORF in other genera, which would be consistent with the diversification hypothesis. Detection of low levels of shorter transcripts arising from the downstream segments of single-ORF *DOA10* orthologs in related hemiascomycetes might provide another test of this idea.

In summary, our study provides clear evidence of an evolutionarily recent eukaryotic gene splitting event that generates a functional two-subunit membrane protein. As the above discussion emphasizes, these data raise a host of interesting questions regarding the evolution and functionality of such proteins compared to the more common single-subunit versions. It should be possible to address many of these issues experimentally.

## Materials and Methods

### Yeast and bacterial methods

Yeast strains and plasmids used in this study are listed in Tables S3 and S4. Standard techniques and methods were used for genetic manipulation of yeast [32] and for recombinant DNA work in *Escherichia coli*.

### Immunoprecipitation and immunoblotting

Cell lysis and protein immunoprecipitation were performed as previously described [33]. After cell lysis the membrane fraction was solubilized in 1% digitonin (SERVA); for immunoprecipitation, anti-HA Agarose (Sigma-Aldrich) was added. Proteins were visualized by immunoblotting. Detailed descriptions are provided in **Information S1**.

### Spot growth assays

Cells were grown to log phase, serially diluted (5-fold) in sterile water and spotted on the indicated media (G418 (Roth); final conc.: 250 µg/ml). Plates were incubated at 30°C for 2–3 days.

### Northern blot analysis

Isolation of total yeast RNA was performed by the hot phenol method as previously described [34]. RNA was separated by electrophoresis through formaldehyde/agarose gels and transferred onto positively charged Nylon membranes (Roche). Membranes were probed with 5′-[^32^P]-labeled oligonucleotide probes in UltraHybOligo hybridization buffer (Ambion). A detailed description is provided in **Information S1**.

## Supporting Information

Figure S1 **FLAG-Nt and Ct-13MYC** ***Kl*** **Doa10 expression levels in transformants from growth assays in** **Fig. 3** **.** A. Lysates of *S. cerevisiae* cells (MHY4175: *doa10Δ*, Deg1-Vma12-KanMX) transformed with expression plasmids for FLAG-tagged *Kl*Doa10 Nt-fragment (FLAG-Nt) and/or 13MYC-tagged *Kl*Doa10 Ct-fragment (Ct-13MYC) or empty plasmids – as indicated - were prepared and processed for immunoblotting with the indicated antibody. In parallel, a lysate from MHY4175 cells expressing *S. cerevisiae* Doa10 from a plasmid (*Sc*Doa10) was analyzed with a Doa10-specific antiserum. Asterisk, nonspecific band. B. Lysates of *S. cerevisiae* (MHY4068; *doa10Δ*, Deg1-URA3) cells transformed with expression plasmids for *Kl*Doa10 FLAG-Nt and/or Ct-13MYC or empty plasmids – as indicated - were prepared and processed as in A). A lysate from MHY4068 cells expressing *S. cerevisiae* Doa10 from a plasmid (*Sc*Doa10) was analyzed with a Doa10-specific antiserum. Asterisk, nonspecific band.
(TIF)

Figure S2 **The 303-bp** ***Kluyveromyces marxianus*** **IVS contains a transcriptional promoter.**A. Schematic representation of the *KmDOA10* gene consisting of Nt-ORF, IVS and Ct-ORF. The annealing sites of Nt- and Ct-ORF specific Northern blot probes are depicted as black horizontal bars. B. Northern blot analysis of *KmDOA10* transcripts with a *KmDOA*10 Nt-ORF specific probe (Nt-probe). Total RNA was isolated from WT *K. lactis* and *K. marxianus* cells and Northern blotting with a *KmDOA*10 Nt-ORF specific probe was carried out as described in Fig. 4. A picture of the stained agarose gel before transfer is shown on the left (gel). The picture on the right shows the signals on the scanned PhosphorImager plate after a 4 d exposure (Nt-probe). M, RNA size markers (in nts). The thin horizontal bar marks the position of the ∼1400-nt *KmDOA10* Nt-ORF specific transcript. No transcript was detected for the *K. lactis* control with the *K. marxianus* specific Nt-probe. C. Northern blot analysis of *KmDOA10* transcripts with a *KmDOA10* Ct-ORF specific probe (Ct-probe). Northern blotting was done as in B., only that the Ct-probe was used instead of the Nt-probe. A picture of the stained agarose gel taken before transfer is shown on the left (gel). The picture on the right shows the signals on the scanned PhosphorImager plate after a 4 d exposure (Ct-probe). M, RNA size markers (in nts). The thin horizontal bar on the right marks the position of the ∼3100-nt *KmDOA10* Ct-ORF specific transcript which is detected for *K. marxianus* but not for *K. lactis*. D. Promoter activity of the *K. marxianus* IVS in absence of additional *KmDOA10* sequences. *Left*: Schematic representation of the *Kl*IVS-URA3-HA, *Km*IVS-URA3-HA and the *Sc*GPD-URA3-HA sequence that were inserted into a promoterless *K. lactis* low-copy plasmid. The constructs were transformed into *K. lactis* cells and expression of the Ura3-HA reporter was examined via anti-HA immunoblotting (*right*). An unspecific background band (bkg) that cross-reacted with pre-immune serum (upon reprobing of the membrane) served as loading control.
(TIF)

Figure S3 **Individual split-** ***Kl*** **Doa10 fragments are stable without the respective partner fragment.** A. Anisomycin-chase analysis of *Kl*Doa10 HA-Nt stability in absence of the *Kl*Doa10 Ct-fragment. The *Kl*Doa10 HA-Nt fragment was expressed in *K. lactis doa10Δ* cells from a low-copy plasmid under control of its original promotor. Following addition of anisomycin, aliquots of cells were taken at the indicated times, and lysates were examined by anti-HA immunoblotting. A background band (bkg) detected after reprobing of the membrane with a pre-immune serum (rabbit) served as a loading control. B. Anisomycin-chase analysis of *Kl*Doa10 Ct-13MYC stability in absence of the *Kl*Doa10 Nt-fragment. The *Kl*Doa10 Ct-13MYC fragment was expressed in *K. lactis doa10Δ* cells from a low-copy plasmid under control of its original promotor ( = IVS sequence). Immunoblotting was done with anti-MYC antibodies. Otherwise as in A. C. Anisomycin-chase analysis of *Kl*Doa10 13MYC-Nt and Ct-13MYC stability upon coexpression of the two fragments. The *Kl*Doa10 13MYC-Nt and Ct-13MYC fragments were coexpressed in *K. lactis doa10Δ* cells from a low-copy plasmid containing the *KlDOA10* locus (with inserted 13MYC-epitope encoding sequences). Immunoblotting was done with anti-MYC antibodies. Otherwise as in A.
(TIF)

Table S1 **Information on fungal Doa10 orthologs listed in** **Fig. 5** **.**
(PDF)

Table S2 **Nucleotide sequences of** ***DOA10*** **intervening sequence in different** ***Kluyveromyces*** **species.**
(PDF)

Table S3 **Yeast strains used in this study, with relevant markers, plasmids and origin of strains.**
(PDF)

Table S4 **Yeast expression plasmids used in this study.**
(PDF)

Information S1 **Supporting Information methods and references.**
(PDF)
