# Supplementary figures and images for: Split-Doa10: A Naturally Split Polytopic Eukaryotic Membrane Protein Generated by Fission of a Nuclear Gene
Source: PLoS One. 2012 Oct 4;7(10):e45194. doi: 10.1371/journal.pone.0045194 (PMC3464245; doi:10.1371/journal.pone.0045194)

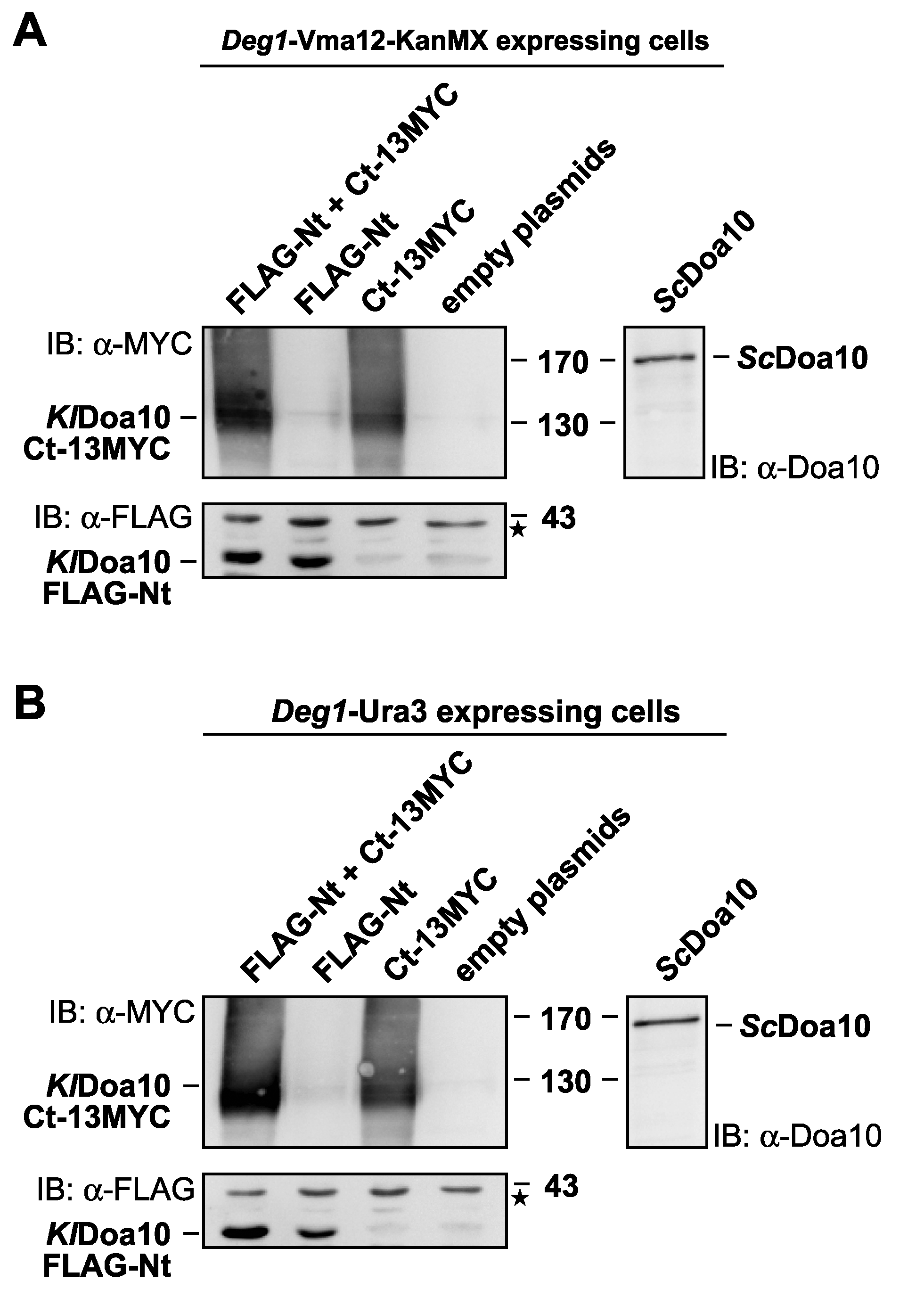

Supplement: Figure S1 — FLAG-Nt and Ct-13MYC Kl Doa10 expression levels in transformants from growth assays in Fig. 3 . A. Lysates of S. cerevisiae cells (MHY4175: doa10Δ, Deg1-Vma12-KanMX) transformed with expression plasmids for FLAG-tagged KlDoa10 Nt-fragment (FLAG-Nt) and/or 13MYC-tagged KlDoa10 Ct-fragment (Ct-13MYC) or empty plasmids – as indicated - were prepared and processed for immunoblotting with the indicated antibody. In parallel, a lysate from MHY4175 cells expressing S. cerevisiae Doa10 from a plasmid (ScDoa10) was analyzed with a Doa10-specific antiserum. Asterisk, nonspecific band. B. Lysates of S. cerevisiae (MHY4068; doa10Δ, Deg1-URA3) cells transformed with expression plasmids for KlDoa10 FLAG-Nt and/or Ct-13MYC or empty plasmids – as indicated - were prepared and processed as in A). A lysate from MHY4068 cells expressing S. cerevisiae Doa10 from a plasmid (ScDoa10) was analyzed with a Doa10-specific antiserum. Asterisk, nonspecific band. (TIF) [file pone.0045194.s001.tif]

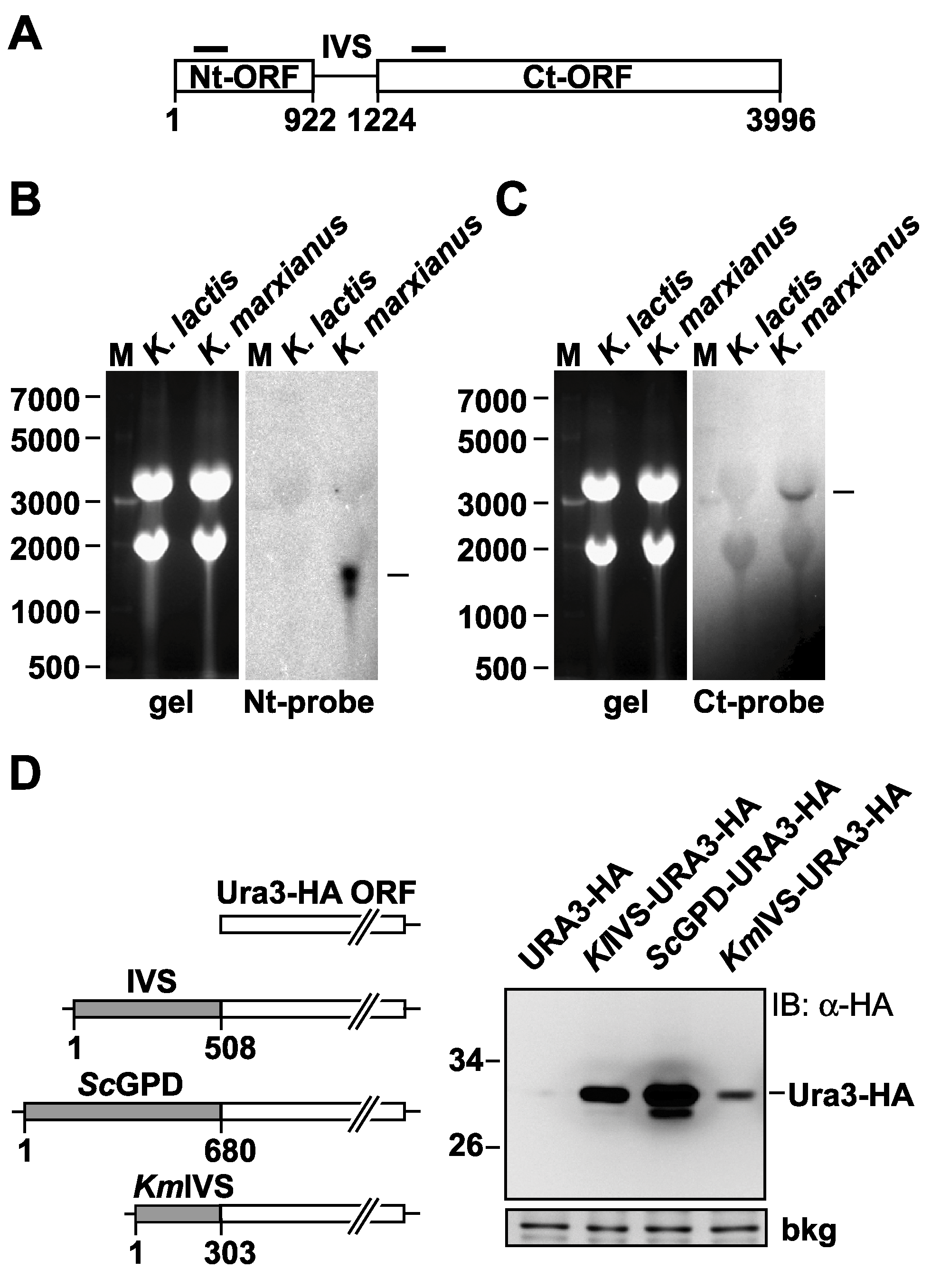

Supplement: Figure S2 — The 303-bp Kluyveromyces marxianus IVS contains a transcriptional promoter.A. Schematic representation of the KmDOA10 gene consisting of Nt-ORF, IVS and Ct-ORF. The annealing sites of Nt- and Ct-ORF specific Northern blot probes are depicted as black horizontal bars. B. Northern blot analysis of KmDOA10 transcripts with a KmDOA10 Nt-ORF specific probe (Nt-probe). Total RNA was isolated from WT K. lactis and K. marxianus cells and Northern blotting with a KmDOA10 Nt-ORF specific probe was carried out as described in Fig. 4. A picture of the stained agarose gel before transfer is shown on the left (gel). The picture on the right shows the signals on the scanned PhosphorImager plate after a 4 d exposure (Nt-probe). M, RNA size markers (in nts). The thin horizontal bar marks the position of the ∼1400-nt KmDOA10 Nt-ORF specific transcript. No transcript was detected for the K. lactis control with the K. marxianus specific Nt-probe. C. Northern blot analysis of KmDOA10 transcripts with a KmDOA10 Ct-ORF specific probe (Ct-probe). Northern blotting was done as in B., only that the Ct-probe was used instead of the Nt-probe. A picture of the stained agarose gel taken before transfer is shown on the left (gel). The picture on the right shows the signals on the scanned PhosphorImager plate after a 4 d exposure (Ct-probe). M, RNA size markers (in nts). The thin horizontal bar on the right marks the position of the ∼3100-nt KmDOA10 Ct-ORF specific transcript which is detected for K. marxianus but not for K. lactis. D. Promoter activity of the K. marxianus IVS in absence of additional KmDOA10 sequences. Left: Schematic representation of the KlIVS-URA3-HA, KmIVS-URA3-HA and the ScGPD-URA3-HA sequence that were inserted into a promoterless K. lactis low-copy plasmid. The constructs were transformed into K. lactis cells and expression of the Ura3-HA reporter was examined via anti-HA immunoblotting (right). An unspecific background band (bkg) that cross-reacted with pre-immune serum ( [file pone.0045194.s002.tif]

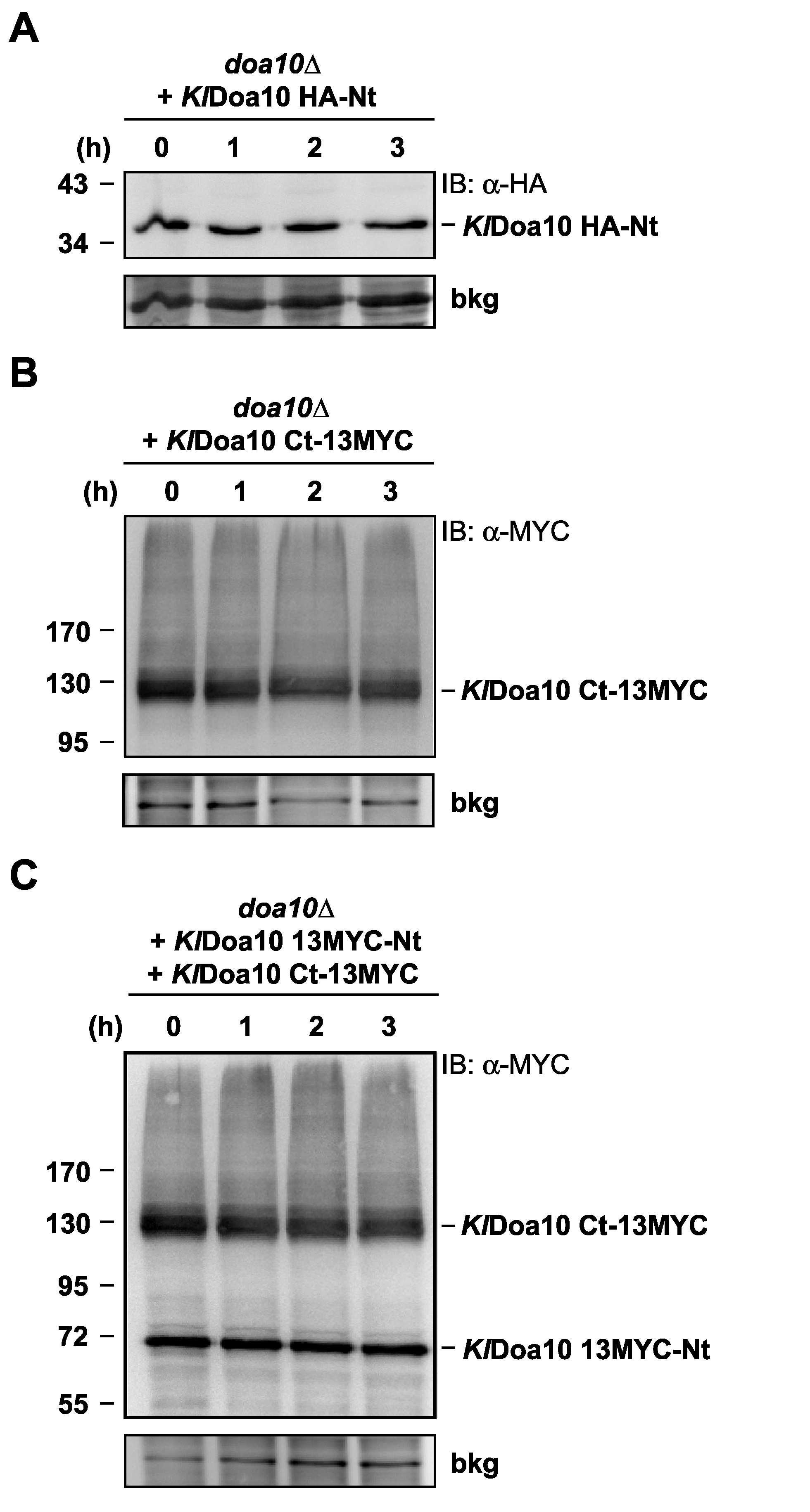

Supplement: Figure S3 — Individual split- Kl Doa10 fragments are stable without the respective partner fragment. A. Anisomycin-chase analysis of KlDoa10 HA-Nt stability in absence of the KlDoa10 Ct-fragment. The KlDoa10 HA-Nt fragment was expressed in K. lactis doa10Δ cells from a low-copy plasmid under control of its original promotor. Following addition of anisomycin, aliquots of cells were taken at the indicated times, and lysates were examined by anti-HA immunoblotting. A background band (bkg) detected after reprobing of the membrane with a pre-immune serum (rabbit) served as a loading control. B. Anisomycin-chase analysis of KlDoa10 Ct-13MYC stability in absence of the KlDoa10 Nt-fragment. The KlDoa10 Ct-13MYC fragment was expressed in K. lactis doa10Δ cells from a low-copy plasmid under control of its original promotor ( = IVS sequence). Immunoblotting was done with anti-MYC antibodies. Otherwise as in A. C. Anisomycin-chase analysis of KlDoa10 13MYC-Nt and Ct-13MYC stability upon coexpression of the two fragments. The KlDoa10 13MYC-Nt and Ct-13MYC fragments were coexpressed in K. lactis doa10Δ cells from a low-copy plasmid containing the KlDOA10 locus (with inserted 13MYC-epitope encoding sequences). Immunoblotting was done with anti-MYC antibodies. Otherwise as in A. (TIF) [file pone.0045194.s003.tif]
