# Supplementary material for: Split-Doa10: A Naturally Split Polytopic Eukaryotic Membrane Protein Generated by Fission of a Nuclear Gene
Source: PLoS One. 2012 Oct 4;7(10):e45194. doi: 10.1371/journal.pone.0045194 (PMC3464245; doi:10.1371/journal.pone.0045194)
Supplement: Table S1 — Information on fungal Doa10 orthologs listed in Fig. 5 . (PDF) [file pone.0045194.s004.pdf]

Table S1. Information on fungal Doa10 orthologs listed in Fig. 5.

Species	GeneID	Length	NCBI Reference Sequence
Saccharomyces cerevisiae [S288c]	GeneID:854781	1319 aa	NP_012234.1
Naumovozyma castelli/ Saccharomyces castelli (CBS 4309)	Gene ID:11529553	1299 aa	XP_003674263.1
Candida glabrata (CBS 138)	GeneID:2889766	1235 aa	XP_447813.1
Zygosaccharomyces rouxii (CBS 732)	GeneID:8204388	1301 aa	XP_002497121.1
Lachancea thermotolerans/ Kluyveromyces thermotolerans (CBS 6340)	Gene ID:8291828	1257 aa	XP_002553679.1
Kluyveromyces lactis [NRRL Y-1140]	GeneID:2894872 (Nt-ORF) GeneID:2894871 (Ct-ORF)	Nt (291 aa) + Ct (923 aa) Σ 1214 aa	Nt-ORF: XP_456313.1 Ct-ORF: XP_456312.1
Kluyveromyces marxianus (DSM No. 70292)	Nt-ORF Ct-ORF	Nt (306 aa) + Ct (923 aa) Σ 1229 aa	GenBank: JQ965807 (Nt-ORF: 1-921; IVS: 922-1224; Ct-ORF: 1225-3996)
Kluyveromyces dobzhanskii (CBS 2104)	Nt-ORF (partial) Ct-ORF (partial)		GenBank: JX036465 (Nt-ORF (5' incomplete): 1-717; IVS: 718-1229: Ct-ORF (3' incomplete): 1230-2106)
Kluyveromyces aestuarii [ATCC 18862]	Nt-ORF Ct-ORF	Nt (284 aa) + Ct (918 aa) Σ 1202 aa	GenBank: AEAS01000184.1 contig00197 (Nt-ORF: 39983-40837; IVS: 40838-41135; Ct-ORF: 41136-43892)
Kluyveromyces wickerhamii [UCD54-210]	Nt-ORF Ct-ORF	Nt (291 aa) + Ct (921 aa) Σ 1212 aa	GenBank: AEAV01000223.1 contig00234 (Nt-ORF: 5293-6168; IVS: 5025-5292; Ct-ORF: 2259-5024 (each on (-)strand))
Eremothecium gossypii/ Ashbya gossypii (ATCC 10895)	GeneID:4620191	1271 aa	NP_984006.1
Candida albicans [SC5314]	GeneID:3703889	1139 aa	XP_888895.1
Yarrowia lioplytica [CLIB122]	GeneID:2908305	977 aa	XP_505617.1
Schizosaccharomyces pombe [972h-]	GeneID:2539855	1242 aa	NP_596733.1
