# Supplementary material for: Split-Doa10: A Naturally Split Polytopic Eukaryotic Membrane Protein Generated by Fission of a Nuclear Gene
Source: PLoS One. 2012 Oct 4;7(10):e45194. doi: 10.1371/journal.pone.0045194 (PMC3464245; doi:10.1371/journal.pone.0045194)
Supplement: Table S2 — Nucleotide sequences of DOA10 intervening sequence in different Kluyveromyces species. (PDF) [file pone.0045194.s005.pdf]

Table S2. Nucleotide sequences of *DOA10* intervening sequence in different *Kluyveromyces* species.

Kluyveromyces lactis IVS (508 bp; NCBI: NC_006042.1: 2569062-2569569 (on (-)strand); genome sequencing: Dujon et al., 2004):

CTATAATCCCCCATTTAAATATTTACGATATTGATAATCTTCATATTGAAACATTGCATCAGCCTTCACTCCAGTTCG
AAATAAACTTCTATATACAATATAATTCCCTCGTTGTCCCATCAAATCATTCACTTGCTTTAGTAAAACATGCTAGCC
CATTAGATTCTCAAAGATGAATTTGGTATTACCCGGCTTATAATGGAAATTGAAATTTGAAGAAGGAAATTTCAACAT
GGGCGCAGACAATCAGTATATACATCGTCGACTGATCTCGTTTCACAACTAAGTACAACAGGTTAACAATATTTAATC
TTTGCTACAATTGTATAGCTGTTGAAGTTGAATAATTATATCATAGAAACTGAATCTTCAACTTGAGTTTTCCGCATT
AGATCCCTGCATTACTCTTTTCTTCTGATCACAATATCATATTTCTTCTGCAACATATTCATATCATTACCAATTAGC
TGAGTTATTAATCGACTGTACTTTTAATTCACAATCCATT

Kluyveromyces marxianus IVS (303 bp; this study; GenBank: JQ965807 (IVS: 922-1224)):

TTAACTCTGACAGCTGTATATATAATATTTGAAATCTTTATGTCTTTATAAATCATGTGCATTCGCTTGCTTTTCCGT
ATACAAAAAGCCTGTAAAACATTTGAAAAAGTGATTACGAAACTCTTTGCAAATTCGTATATAATTTGTAAAAACAAT
AATTATCAACAGTTTGAAAAGAAGGCCTCTTCTACATTCCTATTTAGCTTAATGCTACTCTGAGTATTTGAAAACTGT
GCTGTTGCTCCATATATTACGTTCTTAACCTTGTGTTTTTTGTCTAACTGGAAAGCAGAAGCCTAAAGA

Kluyveromyces aestuarii IVS (298 bp; GenBank: AEAS01000184.1 contig00197 40838-41135; genome sequencing: Baker et al., 2011):

ACATGTTTGCATATTAGTTAGATTTACATTTTTTATGTATCATAATCAAACACTACAAAATTTTGAATATCAGCTAGT
TCAAACACATTCTAATTGTGTTTGCATCCATTTCGATTGTTCACTATGTTGGGTATGCATTCAATTAGGATCAGAACT
GTTTGCCAGTTGATGGTTATTTGGTTACCCGGAAAATAAAATTTTGGACGTACAGATCCTTATGAATTTACTTGCATT
GATTAAAGACCAAACGTGTATTGACTATTCGCATAGGCCGAAGGTTTAGCCATTTCAAAGACCA

Kluyveromyces wickerhamii IVS (268 bp; GenBank: AEAV01000223.1 contig00234 5025-5292 (on (-)strand); genome sequencing: Baker et al., 2011):

ACACTTGCTTCTAGCTTCTCTTCATTGTATAAAGCATCGCACTATTTCATATGTATATGTACCTTCATTCCTTTGTTA
CCCTGACATAGTATATACATATCTAATTTCATAACATGAATTTTCTAATCAATTAGAGAAACGGTTTTATAGAAACAA
GACTGTATAATGACCTTGACCATAGCTATTGTTCTTCATCGCTCACTATATTGTACTTAGCAAGACTCTCTAAGGAAA
ATTTTGAGAAAGAGTAATTGCTGAGAAAAAGAAG

Kluyveromyces dobzhanskii IVS (512 bp; this study; GenBank: JX036465 (IVS: 718-1229)):

TTATTGTTTACTATACAATGTTTAGATTTTGACACTCTACATTAGCTCCACCAGTATAGATTCAAGAAGTTTCTTAGT
ATATAAGGAATAGAGATTTGTTGTGTATGTATGTTGTCTTTGACAATGCTTCTGCAGCCAAACTTTGTGGGGATCACA
ATGAGTTTTCCCACTTACAACTATTGATATTACCCGGCTTTGGGGTAAATTTGAAATCGGAATGAAATTTCAATAGCT
GAATGAATATATCAATACTCCCACCGATCATCAATACTGTACACTCATACAAGTTTATCATTAGCTTCTAAGTACAGC
AGAATATTTTGGATACATTTTCTGCCTCTGATAGCAGAGATCATCGCATTCAGGATTTGTGTTTACCATTAACATCGC
TGCGGTCAACAAATTTACAAAGCGTTTAGTCACACAAGATATTATTCTTGCTCCTAGCATACTCTGCTACTGTTTCTG
AACGAGTAGTTACTTTACTGGTGTATTACTGACCGCTATCTAAC
