# Supplementary material for: Split-Doa10: A Naturally Split Polytopic Eukaryotic Membrane Protein Generated by Fission of a Nuclear Gene
Source: PLoS One. 2012 Oct 4;7(10):e45194. doi: 10.1371/journal.pone.0045194 (PMC3464245; doi:10.1371/journal.pone.0045194)
Supplement: Table S3 — Yeast strains used in this study, with relevant markers, plasmids and origin of strains. (PDF) [file pone.0045194.s006.pdf]

Table S3. Yeast strains used in this study, with relevant markers, plasmids and origin of strains.

Name	Relevant genotype	Source
Kluyveromyces lactis strains		
JA6	*MATα trp1-11 ura3-12 ade1-600 adeT-600*	Breunig & Kuger, 1987
SKY241	JA6 with *doa10Δ::KanMX6*	This study
KHO46-12B (OS162)	*MAT***a** *ura3 leu2 his::loxP*	Heinisch et al., 2010
KHO46-12A (OS163)	*MATα ura3 leu2 his::loxP*	Heinisch et al., 2010
SKY281	KHO46-12A with *doa10Δ::HphMX4*	This study
Other *Kluyveromyces* strains		
DSM 70792	*Kluyveromyces marxianus*; natural isolate	DSM Braunschweig, Germany
CBS 2104	*Kluyveromyces dobzhanskii*; natural isolate	CBS, Utrecht, The Netherlands
Saccharomyces cerevisiae strains		
MHY3000	*MAT***a** *his3-Δ200 leu2-Δ1 ura3-52 lys2-801 trp1-Δ63 doa10-N1-1319myc13-His3MX6 TRP1::pRH373-2HA-UBC7*	T. Ravid and M. Hochstrasser, unpublished data
MHY4086	*MAT***a** *his3-Δ200 ura3-52 trp1-Δ63 leu2-3112 lys2-801::LYS2:: Deg1-URA3 doa10Δ::HphMX4*	This study
MHY4175	*MAT***a** *his3-Δ200 leu2-Δ1 ura3-52 lys2-801 trp1-Δ63 ade2-101 leu2-Δ1::LEU2::pRS305MET25-Deg1-VMA12-KanMX6 doa10Δ::HphMX4*	This study
SKY167	MHY4086 with plasmid YCplac22-GPD-DOA10	This study
SKY272	*MATα his3-Δ200 leu2-Δ1 ura3-52 lys2-801 trp1-Δ63 doa10-1-950myc13-His3MX6 TRP1::pRH373-2HA-UBC7*	This study
SKY330	MHY4175 with plasmid YCplac22-GPD-DOA10	This study
