# Supplementary material for: Split-Doa10: A Naturally Split Polytopic Eukaryotic Membrane Protein Generated by Fission of a Nuclear Gene
Source: PLoS One. 2012 Oct 4;7(10):e45194. doi: 10.1371/journal.pone.0045194 (PMC3464245; doi:10.1371/journal.pone.0045194)
Supplement: Table S4 — Yeast expression plasmids used in this study. (PDF) [file pone.0045194.s007.pdf]

Table S4. Yeast expression plasmids used in this study

Name	Description*	Source
Saccharomyces cerevisiae expression plasmids		
p413GPD	AmpR; *CEN HIS3* with *ScGPD* promoter and *CYCI* terminator	Mumberg et al., 1995
p414MET25	AmpR; *CEN TRP1* with *ScMET25* promoter and *CYCI* terminator	Mumberg et al., 1994
YCplac22	AmpR; *CEN TRP1*	Gietz & Sugino, 1998
p413GPD-FLAG-*Kl*Doa10-Nt	p413GPD with *Kl*Doa10 FLAG-Nt ORF	This study
p413GPD-HA-*Kl*Doa10-Nt	p413GPD with *Kl*Doa10 HA-Nt ORF	This study
YCplac22-GPD-*Kl*Doa10-Ct-13MYC	YCplac22 with *Kl*Doa10 Ct-13MYC ORF	This study
p414MET25-*Kl*Doa10-Ct-13MYC	p414MET25 with *Kl*Doa10 Ct-13MYC ORF	This study
Kluyveromyces lactis expression plasmids		
pCXs18	AmpR; *KlCEN2 ScURA3*	Heinisch et al., 2010
pCse20	AmpR; *KlCEN2 ScLEU2*	Heinisch et al., 2010
KEp6-prom-HA-Nt-ORF-IVS-Ct-ORF-13MYC	AmpR; *2µm URA3* with *Kl*Doa10 HA-Nt ORF and *Kl*Doa10 Ct-13MYC ORF under orig. promoter; *CYCI* terminator; KEp6 backbone (Breunig & Kuger, 1987)	This study
pCXs18-prom-HA-Nt-ORF-IVS-Ct-ORF-HA	pCXs18 with *Kl*Doa10 HA-Nt ORF and *Kl*Doa10 Ct-HA ORF under original promoter; *CYCI* terminator	This study
pCXs18-prom-13MYC-Nt-ORF-IVS-Ct-ORF-13MYC	pCXs18 with *Kl*Doa10 13MYC-Nt ORF and *Kl*Doa10 Ct-13MYC ORF under orig. promoter; *CYCI* terminator	This study
pCXs18-prom-HA-*Kl*Doa10-Nt-ORF	pCXs18 with *Kl*Doa10 HA-Nt ORF under original promoter; *CYCI* terminator	This study
pCXs18-IVS-*Kl*Doa10-Ct-ORF-13MYC	pCXs18 with *Kl*Doa10 Ct-13MYC ORF under original promoter; *CYCI* terminator	This study
pCXs18-IVS(Δ1-150)-*Kl*Doa10-Ct-ORF-13MYC	pCXs18 with *Kl*Doa10 Ct-13MYC ORF preceded by 5' truncated (Δ1-150) *Kl*IVS; *CYCI* terminator	This study
pCXs18-IVS(Δ1-260)-*Kl*Doa10-Ct-ORF-13MYC	pCXs18 with *Kl*Doa10 Ct-13MYC ORF preceded by 5' truncated (Δ1-260) *Kl*IVS; *CYCI* terminator	This study
pCXs18-*Kl*Doa10-Ct-ORF-13MYC	pCXs18 with *Kl*Doa10 Ct-ORF; *CYCI* terminator	This study
pCXs18-URA3-HA	pCXs18 with C-terminally HA tagged Ura3 ORF; *CYCI* terminator	This study
pCXs18-IVS-URA3-HA	pCXs18 with Ura3-HA ORF preceded by *Kl*IVS; *CYCI* terminator	This study
pCXs18-GPD-URA3-HA	pCXs18 with Ura3-HA ORF under *Sc*GPD promoter; *CYCI* terminator	This study
pCXs18-*Km*IVS-URA3-HA	pCXs18 with Ura3-HA ORF preceded by *K.marxianus* IVS; *CYCI* terminator	This study
pCse20-IVS-*Kl*Doa10-Ct-ORF-13MYC	pCse20 with*Kl*Doa10 Ct-13MYC ORF preceded by *Kl*IVS; *CYCI* terminator	This study
pCse20-MET25-Deg1-Vma12-KanMX6	pCse20 with *Deg1*-Vma12-kanMX6 reporter under MET25 promoter; *CYCI* terminator	This study

* Detailed information on generation of plasmids is available from the authors upon request.
