# Supplementary material for: Split-Doa10: A Naturally Split Polytopic Eukaryotic Membrane Protein Generated by Fission of a Nuclear Gene
Source: PLoS One. 2012 Oct 4;7(10):e45194. doi: 10.1371/journal.pone.0045194 (PMC3464245; doi:10.1371/journal.pone.0045194)
Supplement: Information S1 — Supporting Information methods and references. (PDF) [file pone.0045194.s008.pdf]

Supporting Information S1 I

Supporting Information Methods & References

Supporting Information Methods

Yeast and bacterial methods. Yeast rich (YPD) and minimal (SD) medium/plates were prepared as described previously [1]; yeast strains and cultures were grown at 30°C.

Preparation of cell extracts. Cell extracts were prepared as previously described [2]. Briefly, 2.5 A_{600} eq of logarithmically growing cells were lysed by β-mercaptoethanol/NaOH and proteins were precipitated in 5% trichloracetic acid. The pellet was resuspended in SDS-gel loading buffer.

Immunoprecipitation. Cell lysis and immunoprecipitation were performed as previously described [3]. In brief, 25 A_{600} eq of logarithmically growing yeast cells were harvested and resuspended in cold extraction buffer (50 mM Tris, pH 7.5) containing protease inhibitors (625 µM PMSF, 1 µg/ml aprotonin/leupeptin). The cells were disrupted with glass beads by four repeated cycles of vortex mixing for 30 s at maximum speed, interrupted by 30 s incubation on ice. The lysate was diluted with cold extraction buffer and cleared by low-speed centrifugation (2.100 rpm, 5 min). The membrane fraction was collected from the supernatant by centrifugation (13.200 rpm, 10 min) and resuspended in cold resuspension buffer (50 mM Tris, pH 7.5, 200 mM NaOAc, 10% glycerol) containing protease inhibitors (2 µM pepstatin, 1 µg/ml aprotonin/leupeptin and 1 mM Pefabloc). Membranes were solubilized by addition of digitonin (SERVA) to 1% and a 10 min incubation on ice. The digitonin lysate was cleared by centrifugation (13.200 rpm, 10 min) prior to immunoprecipitation. For the immunoprecipitation the digitonin concentration was lowered to 0.5 % by diluting with cold resuspension buffer. An aliquot (5% of total) of the supernatant was removed and served as input for immunoblot. For immunoprecipitation, 10 µl of monoclonal anti-HA agarose beads (Sigma-Aldrich) were added to the digitonin lysate and incubated overnight at 4°C with constant rotation. Beads were collected by low-speed centrifugation, washed four times with cold resuspension buffer, resuspended and heated at 37°C for 15 min in 4x sample buffer (20 mM Tris, pH 6.8, 10% sucrose, 5 mM EDTA, 3% SDS, 10% β-mercaptoethanol, 0.1% bromphenol blue).

Immunoblotting. Proteins were separated by SDS-PAGE and electrotransferred onto a PVDF membrane (Immobilon-P, Millipore). Immunodetection was carried out with appropriate primary antibodies and horseradish peroxidase-conjugated secondary antibodies. Immunoreactive species were visualized using the ECL system (Western Lightning TM Plus-ECL, PerkinElmer). The following antibodies were used: anti-HA mouse monoclonal antibody HA.11 (Covance); anti-c-MYC rabbit polyclonal antibody sc-789 (Santa Cruz Biotechnology); anti-FLAG mouse monoclonal antibody M2 (Sigma). Quantification was carried out by using Aida image analyzer software (Bio Imaging).

Northern blot analysis. Yeast total RNA was prepared by the hot phenol method as described previously [4]. Briefly, cells from a 10 ml YPD culture of 2.5-5.0 A_{600} eq were harvested (3000 rpm, 5 min) and resuspended in 400 µl AE buffer (50 mM NaOAc pH 5.3 , 10 mM EDTA). 40 µl 10% SDS were added and the solution mixed by vortexing. After addition of an equal volume of phenol (equilibrated in AE buffer), samples were vortexed, incubated for 4 min at 65°C and chilled on ice. Following centrifugation (13.200 rpm, 2 min) the aqueous phase was extracted with phenol/chloroform by centrifugation (13.200 rpm, 5 min) at room temperature. RNA was precipitated by addition of 40 µl 3 M NaOAc pH 5.3 and 2.5 volumes of 100% ethanol. The precipitate was washed with 80% ethanol, dried and resuspended in sterile water. For Northern blotting, 25 µg ot total RNA was resuspended to a final concentration of 2 µg/µl in loading buffer (1x MOPS electrophoresis buffer (20 mM MOPS pH 7.0, 2 mM NaOAc, 1 mM EDTA), 50% formamide, 7.4% formaldehyde, 10 µg/ml ethidium bromide). Samples were incubated for 10 min at 65°C, then cooled on ice before loading onto a denaturing 0.9% agarose gel containing 6.6% formaldehyde. Gels were repeatedly washed, first in H_2O, then in 50 mM NaOH followed by 100 mM TrisHCl pH7.0. After soaking for 5 min in 20x SSC (3 M NaCl, 0.3 M NaCitrate pH 7.0) the RNA was transferred by standard capillary transfer in 20x SSC onto a positively charged Nylon membrane (Roche). Membranes were baked for 2 h at 80˚C and prehybridization was carried out for 1 h at 45°C in UltraHybOligo hybridization buffer (Ambion), followed by addition of the 5´-[^{32}P]-labeled oligonucleotide probe (sequences available upon request) and incubation overnight at 42˚C. Membranes were washed twice in washing buffer (2x SSC, 0.5% SDS) and exposed to a BAS reader PhosphorImager (Raytest) or to film.

Anisomycin-chase analysis. *K. lactis* ribosomes are resistent to inhibition by cycloheximide [5]. Therefore, anisomycin was used instead of cycloheximide to inhibit protein synthesis in *K. lactis*.

Anisomycin-chase/Western blot analysis was carried out as described previously [6] except for the use of anisomycin instead of cycloheximide. Briefly, anisomycin (0.15 mg/ml) was added to logarithmically growing *K. lactis* cultures, and cell aliquots were removed at the indicated times after addition. Cells were pelleted, resuspended in cold STOP mix (0.5x SD, 10 mM NaN_3), and stored on ice until all time points had been collected. Lysates were generated, and proteins were visualized by immunoblotting.

***K. lactis doa10*Δ strain generation.** The *K. lactis doa10*Δ strain SKY241 was created by replacement of *DOA10* with a *doa10*Δ::KanMX6 allele in strain JA6 [7] by homologous recombination. Briefly, a ~2.4-kb DNA fragment encompassing the region upstream of the Doa10 ORF (-387 to -1), the KanMX6 marker cassette [8] and the first 520 bp of the Doa10 3'-UTR was transformed into strain JA6 by the LiOAc method [9]. Cells were incubated on a YPD+Ade plate for 16 h and were then replica plated on YPD+Ade plus 300 µg/ml G418. The presence of the *doa10*Δ::KanMX6 allele was verified by PCR. The second *K. lactis doa10*Δ strain, SKY281, was created by replacement of *Doa10* with as *doa10*Δ::HphMX4 allele in strain KHO46-12A (OS163; [10]) similar as described for SKY241 (except for the HphMX4 cassette).

Isolation and sequencing of *K. marxianus* and *K. dobzhanskii DOA10* genes. The *K. marxianus DOA10* locus was amplified with Phusion DNA Polymerase (Finnzyme) from genomic DNA prepared from *K. marxianus* (DSM 70292). Due to the lack of *KmDOA10* sequences in public databases, we first amplified a ~2.5 kb stretch beginning approx. 300 nts upstream of the *Km*Doa10 start codon and ending within the region encoding the highly conserved RPGVL-motif located within the Doa10 TD-domain. The sequence of primer Kons#215 (5'-CCTTCCACTCGTACTTTAGG-3') which anneals approx. 300 bps upstream of the *Km*Doa10 (Nt-)ORF was deduced from a sequence tag (GenBank: AL422796.1) from an exploratory sequencing project of *K. marxianus* by the Génolevures Consortium [11].The degenerated minus strand primer Kons#218 (5'-GACCTDATRAARAANAGAACNCCNGG-3') "matching" the RPGVL-motif encoding sequence was designed based on a multiple alignment of fungal *DOA10* genes. The second half of the *KmDOA10* locus was amplified as part of an approx. 10.5-kb stretch starting just before the RPGVL-motif encoding sequence and ending in the downstream gene locus with similarity to *S.cerevisiae* YER052c. The ~10.5-kb fragment was amplified using primers Kons#231 (5'-GTATTCTCTGTTATCGGTGG-3') annealing upstream of the RPGVL-motif encoding sequence and primer Kons#305 (5-

GGAATGGCACAATAATCTATCCAAGTG-3') annealing approx. 10.5 kb downstream within the ortholog of *S. cerevisiae* YER052c. The sequence of the ortholog of *S. cerevisiae* YER052c (GenBank: AL423471.1) was retrieved from a sequence tag from the same exploratory *K. marxianus* sequencing project as cited above [11], and the distance from the RPGVL-motif encoding *DOA10* sequence was inferred assuming gene synteny between *K. lactis* and *K. marxianus* over that region. Sequencing of the ~2.5-kb fragment as well as sequencing of part of the ~10.5-kb fragment was performed through primer walking. Primer sequences are available upon request.

An approx. 2.1-kb region starting from the RING-CH encoding sequence (approx. 150 bps downstream of the Nt-Doa10 ORF start codon) to the RPGVL-motif encoding sequence was amplified from genomic DNA prepared from *K. dobzhanskii* (CBS 2104). Semi-nested PCR was carried out with the degenerated primer Kons#324 (5'-CCATCCATGYAAGTGYAAAGG-3') designed to anneal within the region encoding the conserved amino acid stretch HPCKCKG within the RING-CH domain of fungal Doa10 and degenerated primers designed to match the RPGVL-motif encoding region (Kons#218 (5'-GACCTDATRAARAANAGAACNCCNGG-3') and nested primer Kons#219 (5'-ATGAARAANAGAACNCCNGGNCK-3'), respectively). Sequencing of the 2.1-kb fragment was performed through primer walking. Primer sequences are available upon request.

Supporting Information References

1. Guthrie C, and Fink, GR (1991) Guide to Yeast Genetics and Molecular Biology; Abelson JN, and Simon, MI, editor. San Diego: Academic Press.
2. Loayza D, Tam A, Schmidt WK, Michaelis S (1998) Ste6p mutants defective in exit from the endoplasmic reticulum (ER) reveal aspects of an ER quality control pathway in Saccharomyces cerevisiae. Mol Biol Cell 9: 2767-2784.
3. Neuber O, Jarosch E, Volkwein C, Walter J, Sommer T (2005) Ubx2 links the Cdc48 complex to ER-associated protein degradation. Nat Cell Biol 7: 993-998.
4. Schmitt ME, Brown TA, Trumpower BL (1990) A rapid and simple method for preparation of RNA from Saccharomyces cerevisiae. Nucleic Acids Res 18: 3091-3092.

5. Dehoux P, Davies J, Cannon M (1993) Natural cycloheximide resistance in yeast. The role of ribosomal protein L41. Eur J Biochem 213: 841-848.
6. Kreft SG, Wang L, Hochstrasser M (2006) Membrane topology of the yeast endoplasmic reticulum-localized ubiquitin ligase Doa10 and comparison with its human ortholog TEB4 (MARCH-VI). J Biol Chem 281: 4646-4653.
7. Breunig KD, Kuger P (1987) Functional homology between the yeast regulatory proteins GAL4 and LAC9: LAC9-mediated transcriptional activation in Kluyveromyces lactis involves protein binding to a regulatory sequence homologous to the GAL4 protein-binding site. Mol Cell Biol 7: 4400-4406.
8. Longtine MS, McKenzie A, 3rd, Demarini DJ, Shah NG, Wach A, et al. (1998) Additional modules for versatile and economical PCR-based gene deletion and modification in Saccharomyces cerevisiae. Yeast 14: 953-961.
9. Gietz RD, Schiestl RH (2007) Quick and easy yeast transformation using the LiAc/SS carrier DNA/PEG method. Nat Protoc 2: 35-37.
10. Heinisch JJ, Buchwald U, Gottschlich A, Heppeler N, Rodicio R (2010) A tool kit for molecular genetics of Kluyveromyces lactis comprising a congenic strain series and a set of versatile vectors. FEMS Yeast Res 10: 333-342.
11. Llorente B, Malpertuy A, Blandin G, Artiguenave F, Wincker P, et al. (2000) Genomic exploration of the hemiascomycetous yeasts: 12. Kluyveromyces marxianus var. marxianus. FEBS Lett 487: 71-75.
